# Evaluating the diagnostic and prognostic value of serum TuM2‐PK, NSE, and ProGRP in small cell lung cancer

**DOI:** 10.1002/jcla.24865

**Published:** 2023-04-23

**Authors:** Li Li, Qian Zhang, Yuchao Wang, Chunhua Xu

**Affiliations:** ^1^ Department of Respiratory Medicine, Affiliated Nanjing Brain Hospital Nanjing Medical University Nanjing Jiangsu 210029 China; ^2^ Clinical Center of Nanjing Respiratory Diseases and Imaging Nanjing Jiangsu 210029 China

**Keywords:** diagnosis, neuron‐specific enolase, progastrin‐releasing peptide, prognosis, small cell lung cancer, tumor M2‐pyruvate kinase

## Abstract

**Background:**

The aim of this study was to explore the diagnostic and prognostic value of serum tumor M2‐pyruvate kinase (TuM2‐PK), neuron‐specific enolase (NSE), and progastrin‐releasing peptide (ProGRP) levels in patients with small cell lung cancer (SCLC).

**Methods:**

The levels of serum TuM2‐PK, NSE, and ProGRP in 102 patients with SCLC, 60 patients with benign lung disease (BLD), and 90 healthy controls were detected.

**Results:**

The serum TuM2‐PK, NSE, and ProGRP levels in the SCLC group were higher than those in BLD group (*p* < 0.05) and healthy control group (*p* < 0.05). The sensitivity of TuM2‐PK, NSE, and ProGRP detection in SCLC was 82.35%, 60.78%, and 77.45% respectively, and specificity was 91.11%, 81.11%, and 86.67%, respectively. The area under the curve (AUC) of SCLC resulting from TuM2‐PK was significantly better than that of NSE and ProGRP. The application of TuM2‐PK combined with NSE and ProGRP improved the diagnostic yield of SCLC patients and had better diagnostic value than TuM2‐PK alone. Univariate and multivariate analysis indicated that an elevated TuM2‐PK level was an independent prognostic factor for shorter survival in SCLC.

**Conclusions:**

These results suggest that TuM2‐PK levels in the serum could be an effective biomarker for the diagnosis and prognosis of SCLC.

## Graphical abstract

To explore the diagnostic and prognostic value of serum TuM2‐PK, NSE, and ProGRP levels in SCLC patients. Serum TuM2‐PK, NSE, and ProGRP levels were higher in the SCLC group than the controls group. Univariate and multivariate analysis indicated that an elevated TuM2‐PK level was an independent prognostic factor for shorter overall survival in SCLC. These results suggest that TuM2‐PK levels in the serum could be a new effective biomarker for the diagnosis and prognosis of SCLC.

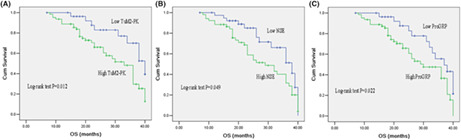

## 1 INTRODUCTION

Lung cancer is one of the most common malignancies in the world, accounting for 13% of all cancers.^1^ SCLC accounts for 15%–18% of all lung cancers.^2^ More than half of SCLC patients are in the extensive stage of the disease at the time of diagnosis. Despite systematic treatment, the overall 5‐year survival rate for SCLC remains below 15%.^3^ Therefore, it is necessary to seek novel biomarkers in order to diagnose and predict the progress of SCLC. The ProGRP and NSE are common tumor markers in the diagnosis of tumor.^4^, ^5^ However, these widely used blood markers are not accurate enough for diagnosis. Therefore, the search for new biomarkers remains an important part of SCLC diagnosis.

TuM2‐PK is an important glycolytic process enzyme that plays a role in tumor metabolism. The increased expression of TuM2‐PK in many tumor patients is associated with the pathogenesis of tumor.^6^, ^7^, ^8^, ^9^, ^10^, ^11^ Previous study has shown an increase in plasma TuM2‐PK levels in lung cancer patients.^12^ Study has shown that the use of TuM2‐PK in the diagnosis of SCLC can improve the diagnosis yield.^13^ However, the relationship between blood TuM2‐PK and the progression of SCLC and the effect of TuM2‐PK on the SCLC survival have not been fully studied.

The purpose of this study was to analyze the levels of serum TuM2‐PK, NSE, and ProGRP in SCLC patients and to evaluate their clinical value in the diagnosis and prognosis of SCLC.

## 2 MATERIALS AND METHODS

### 2.1 Patients

From January 2017 to April 2020, we prospectively enrolled 102 patients who attended the Nanjing Brain Hospital, affiliated to the Nanjing Medical University for the primary treatment of SCLC. The patients included 55 (53.9%) males and 47(46.1%) females, with a median age of 55 years. All SCLC were diagnosed cytologically or histologically by a pathologist. Patients with SCLC were included if they met the following criteria: confirmation of SCLC via a review of pathologic slides by two independent observers to classify the histologic subtype; no pro‐surgical or pro‐diagnostic history of antineoplastic therapy, radiotherapy, or chemotherapy. The SCLC patients were staged according to the Veterans Administration Lung Cancer Research Group (VASG) staging system.^14^ Limited disease was defined as disease confined to one hemithorax including the mediastinal lymph nodes and/or the supraclavicular lymph nodes; extended disease was defined as having limited disease or malignant pleural effusion. Sixty BLD patients (35 men and 25 women, median age 52) and 90 healthy volunteers (48 men and 42 women, median age 54) were included in the control group in the same period. Patients with BLD were identified via CT screening, etiology, and response to antibiotics and subsequently monitored for 6 months using CT, with no evidence of cancer. None had a history of previous cancer or chemotherapy. Healthy volunteers were subjects who had not received a diagnosis of malignant or benign disease after routine examinations, including CT, ultrasonographic examination, and routine laboratory tests. All SCLC patients received combination chemotherapy based on cisplatin or carboplatin for at least two cycles of first‐line treatment. The clinicopathological features of patients were collected. Follow‐up information was obtained by phone or Wechat. The deadline for follow‐up is March 21, 2021. Overall survival (OS) refers to the time between diagnosis date and the death date or the last follow‐up.

The study was approved by the Ethics Committee of the Nanjing Brain Hospital, affiliated to the Nanjing Medical University (2017, Number: NJ20170314). All patients expressed informed consent.

### 2.2 Measurement of serum TuM2‐PK, NSE, and ProGRP levels

After diagnosis, 10 mL blood samples were taken from each patient before treatment. The sample was centrifuged at 1000 *g* for 15 min, and the supernatant was immediately stored at 80°C until use. The concentrations of NSE and ProGRP were determined by electrochemiluminescence (R&D Systems). The TuM2‐PK concentration in serum was determined by ELISA according to the instructions of the manufacturer. A monoclonal antibody specific for the dimeric TuM2‐PK and with no cross‐reaction with the other pyruvate kinase isoenzymes (ScheBo Tech) was used. All assays were performed in duplicate. The assay allows quantification of TuM2‐PK within the range of 5–100 U/mL. The reference concentration of TuM2‐PK is <15 U/mL. Values in the range of 15–20 U/mL are of questionable importance. The intra‐assay coefficient of variance was 3.5%, and the inter‐assay variance was 5.3%. Each sample in duplicate, take the average value. The technicians did not know anything about clinical data. The normal value range is NSE: 0–20 ng/mL and ProGRP: 0–65 pg/mL.

### 2.3 Statistical analysis

Data analysis used the statistical software (SPSS for Windows, version 18). All values are given in the form of mean ± standard deviation. The Mann–Whitney *U* test was used to compare the differences among serum samples. The ROC curve was used to evaluate the diagnostic value of serum marker, and the AUC was calculated. The OS was evaluated by Kaplan–Meier method and Cox regression analysis. Cox proportional hazard model was used to calculate the hazard ratio and the corresponding 95% confidence interval (CI). *p* < 0.05 was statistically significant.

## 3 RESULTS

### 3.1 Patients characteristics

Of the 102 SCLC patients, 55 were men and 47 were women, with a median age of 55 years (range 35–76 years). There were 63 smokers and 39 non‐smokers. Forty‐two patients were limited SCLC, and 60 patients were extended SCLC stages. Ninety‐two patients received etoposide and cisplatin chemotherapy, and 71 received radiotherapy. After treatment, 69 patients achieved complete or partial remission, while 33 patients remained stable or progressed. The median follow‐up was 12 months (ranging from 6 to 36 months), median OS was 15 months, limited SCLC was 18 months, and extended SCLC was 10.5 months.

### 3.2 Serum TuM2‐PK, NSE, and ProGRP levels in SCLC patients

**FIGURE 1:**
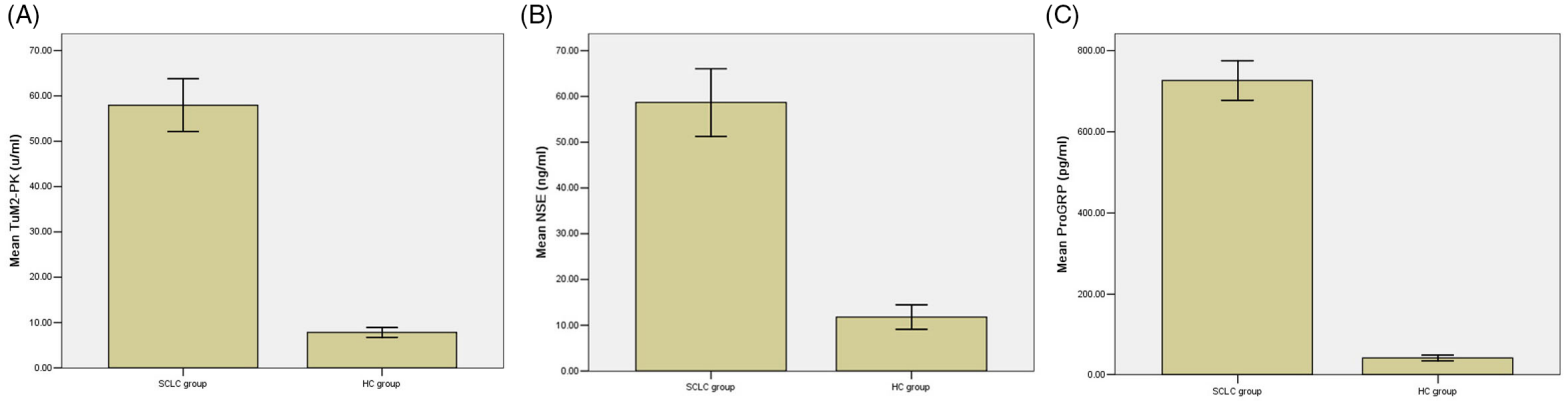
Serum levels of TuM2‐PK, NSE, and ProGRP in SCLC patients. Among 102 SCLC patients, the serum levels of TuM2‐PK (A), NSE (B), and ProGRP (C) were significantly higher than those of BLD group and healthy control group (*p* < 0.05). NSE, neuron‐specific enolase; ProGRP, progastrin‐releasing peptide; SCLC, small cell lung cancer; TuM2‐PK, tumor M2‐pyruvate kinase.

The levels of serum TuM2‐PK in SCLC group were significantly higher than those in BLD group and healthy control group (57.93 ± 10.51 U/mL vs. 12.79 ± 2.18 U/mL vs. 7.83 ± 1.85 u/ml, *p* < 0.05) (Figure 1A). Similarly, the levels of serum NSE and ProGRP were also higher in SCLC patients than those in BLD group and healthy control group (58.69 ± 13.35 ng/mL vs. 15.08 ± 2.43 ng/mL vs. 11.86 ± 0.77 ng/mL, *p* < 0.05; 726.95 ± 89.84 pg/mL vs. 72.33 ± 25.66 pg/mL vs. 41.43 ± 6.57 pg/mL, *p* < 0.05) (Figure 1B,C).

### 3.3 Diagnostic value of TuM2‐PK, NSE, and ProGRP

**FIGURE 2:**
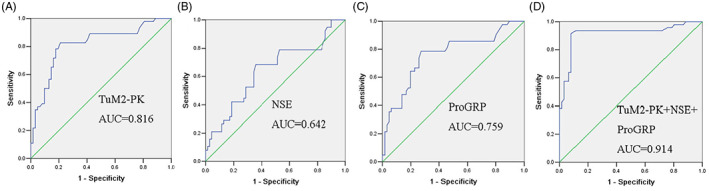
Comparison of TuM2‐PK, NSE, ProGRP, and their combination on diagnosis for SCLC. ROC analysis resulted in an AUC of 0.816, 0.642, 0.759, and 0.914 for TuM2‐PK (A), NSE (B), ProGRP (C), and TuM2‐PK + NSE + ProGRP (D), respectively. NSE, neuron‐specific enolase; ProGRP, progastrin‐releasing peptide; SCLC, small cell lung cancer; TuM2‐PK, tumor M2‐pyruvate kinase.

TuM2‐PK was calculated to differentiate the sensitivity between SCLC and the control. As shown in Figure 2A, the AUC of TuM2‐PK curve was 0.816. When the cutoff was 50.18 U/mL, TuM2‐PK had 82.35% sensitivity and 91.11% specificity to differentiate SCLC from the controls. Serum TuM2‐PK is an effective biomarker for the diagnosis of SCLC.

The efficiency of NSE and ProGRP in differentiating SCLC from the controls was summarized in Table 1. The AUC for SCLC resulting from TuM2‐PK was significantly superior to NSE and ProGRP (Figure 2B,C and Table 1). These results suggest that serum TuM2‐PK was a valuable biomarker for the diagnosis of SCLC.

**TABLE 1:** Diagnostic value of serum levels of TuM2‐PK, NSE, ProGRP, and their combination in SCLC patients.

SCLC vs. HC	AUC (95% CI)	Sensitivity (%)	Specificity (%)	Accuracy (%)	PPV (%)	NPV (%)
TuM2‐PK	0.816 (0.731–0.901)	82.35	91.11	86.64	91.30	82.00
NSE	0.642 (0.529–0.755)	60.78	81.11	70.31	78.48	64.60
ProGRP	0.759 (0.662–0.856)	77.45	86.67	81.77	86.81	77.23
TuM2‐PK + NSE + ProGRP	0.914 (0.851–0.977)	89.22	88.89	89.06	90.10	87.91

Abbreviations: AUC, areas under the curves; HC, healthy controls; NPV, negative predictive value; PPV, positive predictive value; SCLC, small cell lung cancer.

The application of TuM2‐PK combined with NSE and ProGRP improved the diagnostic yield of SCLC patients and had better diagnostic value than TuM2‐PK alone (Figure 2D).

### 3.4 Relationship between TuM2‐PK, NSE, and ProGRP levels and clinicopathological features

The levels of serum TuM2‐PK were significantly correlated with disease stage (*p* = 0.013), but not with age (*p* = 0.267), sex (*p* = 0.365), smoking (*p* = 0.189), and ECOG performance status (*p* = 0.538). The levels of serum NSE and ProGRP correlated with disease stage (Table 2).

**TABLE 2:** Comparison of serum TuM2‐PK, NSE, and ProGRP levels depending on clinical characteristics in SCLC patients.

Variables	TuM2‐PK (U/mL)	*p*	NSE (ng/mL)	*p*	ProGRP (pg/mL)	*p*
Age (year)						
≥60	57.13 ± 11.23	0.267	58.54 ± 15.36	0.138	688.36 ± 85.46	0.467
<60	58.64 ± 10.98		57.61 ± 15.97		730.45 ± 79.59	
Sex						
Male	59.04 ± 9.73	0.365	55.68 ± 14.68	0.316	730.42 ± 87.53	0.318
Female	55.27 ± 12.65		59.12 ± 15.27		715.65 ± 88.15	
Smoking status						
Nonsmoker	56.46 ± 11.68	0.189	59.01 ± 14.84	0.132	805.18 ± 92.32	0.069
Smoker	58.87 ± 13.38		56.43 ± 14.68		670.34 ± 86.83	
Performance status						
0–1	57.61 ± 15.88	0.538	58.35 ± 14.87	0.245	792.64 ± 95.52	0.367
2–3	56.75 ± 10.61		56.52 ± 14.67		619.25 ± 99.26	
Disease stage						
Limited	36.67 ± 12.34	0.013*	32.37 ± 15.64	0.001*	515.37 ± 89.27	0.003*
Extended	78.53 ± 10.45		75.46 ± 16.56		839.53 ± 86.78	

*Note*: The applied statistical method was Mann–Whitney *U* test.

Abbreviations: NSE, neuron‐specific enolase; ProGRP, progastrin‐releasing peptide; TuM2‐PK, tumor M2‐pyruvate kinase.

* Significant difference.

### 3.5 Correlation of serum TuM2‐PK, NSE, and ProGRP levels with overall survival

**FIGURE 3:**
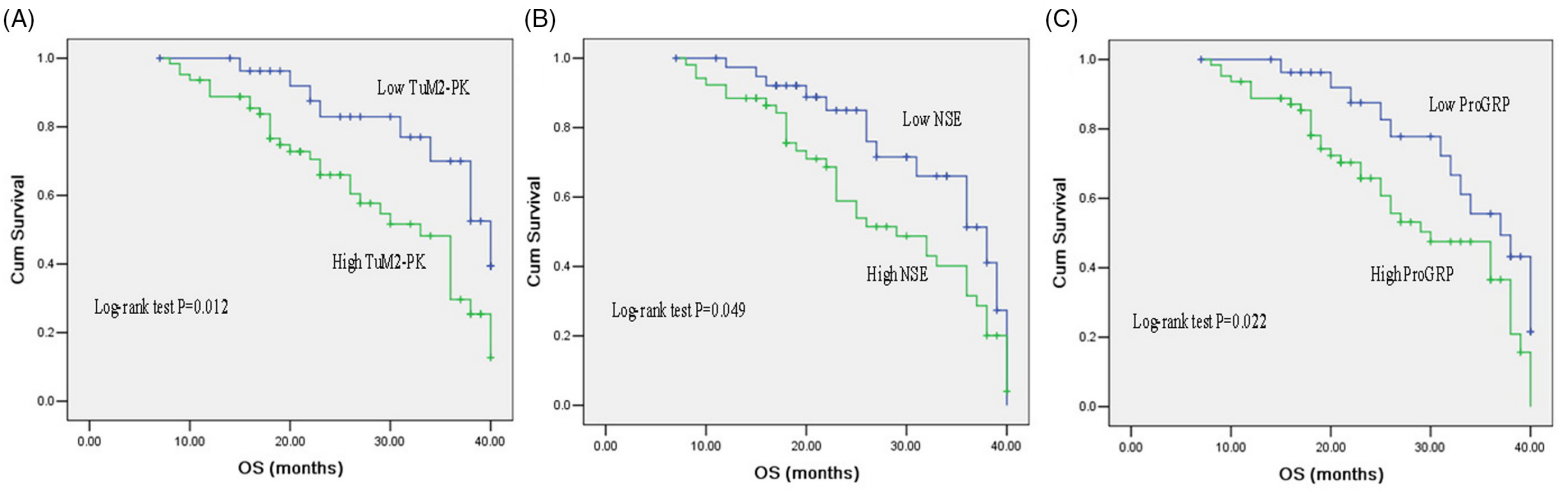
Kaplan–Meier survival analysis of SCLC patients based on serum TuM2‐PK, NSE, and ProGRP levels. The overall survival of SCLC patients with high serum TuM2‐PK level, NSE level, and ProGRP level was significantly lower than patients with low serum TuM2‐PK level (A), NSE level (B), and ProGRP level (C). NSE, neuron‐specific enolase; ProGRP, progastrin‐releasing peptide; SCLC, small cell lung cancer; TuM2‐PK, tumor M2‐pyruvate kinase.

To evaluate the correlation between TuM2‐PK, NSE, and ProGRP levels and survival, patients were divided into high‐level and low‐level groups according to the cutoff values of TuM2‐PK, NSE, and ProGRP. The results of OS analysis showed that compared with the patients with lower serum TuM2‐PK level, NSE level, and ProGRP level, the patients with higher serum TuM2‐PK level, NSE level, and ProGRP level had shorter OS (Figure 3A–C).

Univariate analysis showed that tumor stage (*p* = 0.001), TuM2‐PK (*p* = 0.015), NSE (*p* = 0.003), and ProGRP (*p* = 0.001) were significantly associated with prognosis. Multiple regression analysis showed that stage (*p* = 0.026) and TuM2‐PK (*p* = 0.003) were independent prognostic factors for OS (Table 3).

**TABLE 3:** Univariate and multivariate Cox analysis of variables considered for OS of SCLC patients.

Characteristics	Univariate			Multivariate		
	HR	95% CI	*p*	HR	95% CI	*p*
Sex (male vs. female)	1.326	0.571–2.962	0.674	1.109	0.915–1.342	0.291
Age (<60 vs. ≥60)	1.252	0.561–2.794	0.583	1.724	0.336–2.896	0.754
Disease stage (limited vs. extended)	4.685	1.833–11.974	0.001*	4.125	1.182–14.396	0.026*
Performance status (0–1 vs. 2–3)	0.966	0.913–1.022	0.226	0.947	0.429–2.090	0.892
Smoking history (ever vs. never)	0.659	0.375–1.157	0.147	0.898	0.704–1.144	0.383
TuM2‐PK (≥15 U/mL vs. <15 U/mL)	3.017	1.242–7.328	0.015*	3.278	1.486–7.231	0.003*
NSE (≥20 ng/mL vs. <20 ng/mL)	1.131	1.025–1.237	0.003*	0.627	0.381–1.032	0.067
ProGRP (≥65 pg/mL vs. <65 pg/mL)	1.540	1.090–1.991	0.001*	1.319	0.734–2.369	0.354

Abbreviations: CI, confidence interval; HR, hazard ratio; OPN, osteopontin; OS, overall survival.

* Significant difference.

## 4 DISCUSSION

Small cell lung cancer often metastasizes in the early stage because of its low differentiation, high malignancy, and rapid growth.^15^, ^16^ Since most SCLC patients were locally advanced or had distant metastasis at the time of initial treatment, they lost the opportunity for operation. At the same time, because SCLC is highly sensitive to radiotherapy and chemotherapy, the most common treatment strategy is the comprehensive treatment scheme based on chemotherapy.^17^ Therefore, the prevention and treatment of SCLC should focus on early diagnosis, reasonable individualized treatment, and accurate assessment of prognosis.

TuM2‐PK is a clinically valuable tumor marker, which has been applied in the diagnosis, evaluation of curative effect, and evaluation of prognosis.^18^, ^19^ The expression of TuM2‐PK in normal human serum was low, but increased in tumor state. The level of serum TuM2‐PK level in SCLC patients was significantly higher than that in the controls and BLD patients, which suggested that TuM2‐PK may play an important role in the development of lung cancer and has become a new tumor marker for diagnosis and prognosis. Our previous findings showed that serum TuM2‐PK level was higher in NSCLC patients than in the controls.^20^

Schneider et al. found that TuM2‐PK was the best single indicator for detection SCLC, with a specificity of 90%, while NSE was only 32%. The combined detection of TuM2‐PK, NSE, and ProGRP can increase the sensitivity to 67%. When the two markers were combined, the best combination was ProGRP and TuM2‐PK, and the sensitivity was 56%.^21^ In this study, TuM2‐PK was a valuable marker for the diagnosis of SCLC, with sensitivity and specificity of 82.35% and 91.11%, respectively. To further evaluate the potential of TuM2‐PK as a diagnostic marker for SCLC, we compared it with ProGRP and NSE. Compared with control group, the AUC value of TuM2‐PK in SCLC was higher than that of ProGRP and NSE. We also compared the diagnostic efficacy of ProGRP and NSE with that of TuM2‐PK. The results showed that the combined detection of these three markers was superior to the single marker detection in the diagnosis of SCLC. This may provide a new way to diagnose SCLC.

A previous study showed an association between TuM2‐PK and tumor cell progression.^21^ In our study, we found a correlation between serum TuM2‐PK levels and staging, suggesting that the increased serum TuM2‐PK levels may result in tumor cells. In addition, our results suggest that elevated serum TuM2‐PK can predict poor OS in SCLC patients.

Several limitations of our study warrant discussion. First, we performed the study at a single center with relatively small sample size. Second, the expression of TuM2‐PK in serum of lung cancer patients was detected, but the expression of TuM2‐PK in lung cancer tissues was not detected. Third, the specific mechanism of the relationship between TuM2‐PK expression and SCLC was lacking. Further perspective trial should be performed.

In conclusion, we demonstrated elevated serum TuM2‐PK levels in SCLC patients. TuM2‐PK levels were associated with survival in SCLC patients. These results suggest that serum TuM2‐PK may be an effective biomarker for SCLC diagnosis and prognosis. Further research is needed to clarify the mechanism of TuM2‐PK in tumorigenesis.

## CONFLICT OF INTEREST STATEMENT

The authors declare not any conflicts of interest in this work.

## Data Availability

The data that support the findings of this study are available from the corresponding author upon reasonable request.
